# Universal public use of surgical mask and respiratory viral infection

**DOI:** 10.1002/jgf2.340

**Published:** 2020-05-29

**Authors:** Kazuhiro Kamata, Norio Ohmagari, Yasuharu Tokuda

**Affiliations:** ^1^ Infectious Diseases Research Center of Niigata University Yangon Myanmar; ^2^ Disease Control and Prevention Center National Center for Global Health and Medicine Hospital Tokyo Japan; ^3^ Muribushi Okinawa Center for Teaching Hospitals Okinawa Japan

During the COVID‐19 pandemic, the World Health Organization warned the shortage of surgical masks because of the increased demanding and stocking up.^1^ The price of masks has surged in many countries. In Japan, the practice of wearing a mask has been common not only during this COVID‐19 outbreak but also during winter seasons every year, although it has been unclear whether wearing a mask universally could reduce the risk of respiratory viral infection in a population.^2^ Feng et al recently suggested that rational recommendations on appropriate face mask use should be developed.^3^

A nationwide online cross‐sectional survey was conducted to assess the effectiveness of mask use among the general public in Japan in March 2017. Based on the panel of 7.6 million people registered with a research company, the survey participants were selected from adults aged 20‐69 years who were not medical professionals. People aged 70 years or older were excluded because of their potential difficulties in responding to the online survey. Additionally, the participants were selected to reflect the distribution of the population (national population census of Japan in 2015) with regard to gender, age, place of residence (prefecture), and population size.^4^

In a total of 3390 responses, 64% reported a habitual practice of wearing a surgical mask during all winter seasons over the last 5years. During these winter seasons, 60% of participants reported at least single episode of symptoms of respiratory viral infection. Among those who wore a mask, 65% reported the development of the episodes. Among those who did not, 50% reported its development. Thus, the greater proportion of the episodes were identified in mask‐wearing people.

Our results indicate that there may be little protective effects of habitual use of a surgical mask on experiencing episodes of respiratory viral infection during winter seasons. A meta‐analysis on the published literature showed, on the other hand, that infected patients wearing a mask could reduce risk of infecting others.^5^ Our findings may have limitations including a recall bias because of the self‐reports. However, governments and healthcare providers may need to educate people that little evidence exists about surgical mask use among the general public for receiving respiratory viral infection and it is likely that its use among infected patients, including presymptomatic or asymptomatic patients with COVID‐19, could lower possibility of infecting others.

## CONFLICT OF INTEREST

The authors have stated explicitly that there are no conflicts of interest in connection with this article.
